# On the impact of smart sensor approximations on the accuracy of machine learning tasks

**DOI:** 10.1016/j.heliyon.2020.e05750

**Published:** 2020-12-16

**Authors:** Daniele Jahier Pagliari, Massimo Poncino

**Affiliations:** Dipartimento di Automatica e Informatica (DAUIN), Politecnico di Torino, Corso Duca degli Abruzzi 24, 10129, Turin (TO), Italy

**Keywords:** Computer science, Machine learning, Energy efficiency, Approximate computing

## Abstract

Smart sensors present in ubiquitous Internet of Things (IoT) devices often obtain high energy efficiency by carefully tuning how the sensing, the analog to digital (A/D) conversion and the digital serial transmission are implemented. Such tuning involves *approximations*, i.e. alterations of the sensed signals that can positively affect energy consumption in various ways. However, for many IoT applications, approximations may have an impact on the quality of the produced output, for example on the classification accuracy of a Machine Learning (ML) model. While the impact of approximations on ML algorithms is widely studied, previous works have focused mostly on *processing* approximations.

In this work, in contrast, we analyze how the signal alterations imposed by smart sensors impact the accuracy of ML classifiers. We focus in particular on data alterations introduced in the serial transmission from a smart sensor to a processor, although our considerations can also be extended to other sources of approximation, such as A/D conversion. Results on several types of models and on two different datasets show that ML algorithms are quite resilient to the alterations produced by smart sensors, and that the serial transmission energy can be reduced by up to 70% without a significant impact on classification accuracy. Moreover, we also show that, contrarily to expectations, the two generic approximation families identified in our work yield similar accuracy losses.

## 1 Introduction

The explosive growth of machine learning (ML) algorithms, especially based on deep neural networks (DNNs) is expected to enhance a wide range of Internet of Things (IoT) applications, ranging from activity tracking to embedded natural language processing and computer vision [1]. These algorithms are originally developed to run on powerful GPU-based servers on the cloud. However, in many application domains, it is desirable to implement them locally on the IoT devices (i.e. at the “edge”) [2], [3]. This eliminates the need of transmitting raw data to the cloud, typically through a wireless link, and can therefore yield several benefits, including:

• A reduced and more predictable response latency in presence of slow or intermittent connectivity
• An improved security for the user since private raw data never leave the device
• A reduced energy consumption, since wireless transmission is a very energy-hungry operation

Despite these promises, implementing ML algorithms on IoT edge devices are not an easy task. Besides the well known limitations in terms of processing speed and memory space [2], another issue is related to the limited energy budget of IoT devices, which are typically battery-operated and expected to operate for months or years without recharging [4]. Running on battery for such a long time requires an optimal management of the available energy in *all* phases of operation of an IoT edge device. Therefore, all subsystems are optimized for energy, including sensing, Analog-to-Digital (A/D) and Digital-to-Analog (D/A) conversion, processing, actuation and data transmission [1], [5]. Such energy-optimized components often obtain high efficiency by means of various forms of *data approximations* [6], [7], [8], [9], [10], [11], [12], [13], [14]. They are, in other words, designed according to the so-called *Approximate Computing* paradigm, which has recently gained a lot of traction in both academia and industry [15], [16], [17]. The impact of approximate computing strategies on ML algorithms has been widely studied in literature [2], [3], [18], [19]. However, the great majority of focus has been devoted only to *processing* approximations [20], [21], [22], [23], [24], [25], [26], [27], [28], [29].

In this work we take on a different and novel perspective, namely, we assess the impact of the energy-driven approximations in the *data acquisition* path on the quality of the ML algorithms. Specifically, we focus on the approximated transmission of data from a smart sensor to a processor, through energy-efficient bus encodings [7], [8], [9], [10], [11]. Besides being relevant per se, due to the large amount of energy consumed by off-chip sensor-processor connections [10], these approximations are also similar to (and therefore representative of) other data alterations that can appear in data acquisition chains, such as adaptive sampling frequency [6], [30], [31] and adaptive A/D conversion [32], [33]. Therefore, the analysis of serial transmission also provides relevant insights on other elements of the chain.

In our experiments, we assess the effectiveness of three smart sensor transmission approximations on two edge ML tasks, i.e. activity recognition based on Inertial Measurement Units (IMUs) and image classification. The approximations considered are based on opposing underlying principles, which we denote as *smoothing* and *rounding*. Results show that ML classification algorithms are in general quite resilient to the alterations produced by smart sensors. Energy reductions up to 70% can be obtained on both target tasks, without a significant impact on classification accuracy. Moreover, we also show that approximations that, contrary to expectations, approximations based on the *smoothing* and *rounding* principles yield comparable results and that the latter actually have the potential to reach even greater savings for a given accuracy level.

The rest of the paper is structured as follows. Section 2 describes the types of approximations that can be implemented by a smart sensor and their impact on energy consumption. Section 3 then focuses specifically on serial transmission approximations, which are the main focus of this work, while Section 4 analyzes related research on the impact of approximations on ML tasks. Finally, Section 5 contains experimental results and Section 6 concludes the paper.

## 2 Smart sensors and approximation

### 2.1 Smart sensor operations

**Figure 1:**
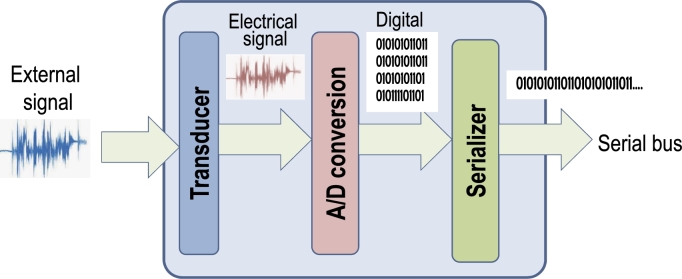
Conceptual block diagram of a sensor.

A generic smart sensor consists of three main elements as shown in Fig. 1.

The *transducer* constitutes the interface with the external world, and translates the environmental signal (light, sound, vibration, temperature, etc) into the electrical domain. The transducer typically includes also some circuitry for *signal conditioning*, such as the adjustment of the signal bias and magnitude (via amplification) to match the requirements of the downstream components. Moreover, conditioning typically includes circuitry to “clean” the signal through various types of *filtering* that depend on the characteristics of the signal itself.

The conditioned and filtered analog electrical signal is periodically sampled and converted to the digital domain by an Analog to Digital (A/D) converter. In the most common scenario (uniform quantization), the digital value is assigned proportionally to the ratio between the sampled analog value and a reference value, in the range from 0 to $2^{n}-1$ where *n* is the resolution of the converter, i.e. the number of bits in the output code.

Digital samples are then transferred to the processing part of the system as data to be used for the computation. The de-facto standard is to transfer these data *serially*, using standard protocols such as *I*^*2*^*C*, *SPI* or *CAN* [34]. Serial links are preferred to parallel ones for several reasons, such as the reduced skew and jitter issues, which allow larger transmission frequencies, the reduced pin count and wire area, and the easier routing layout on a Printed Circuit Board (PCB).

Approximations are possible in each of the three blocks described above, as summarized in Table 1.

**Table 1:** Knobs for signal approximation in a sensor.

Block	Operation	Alteration
Transducer	Sensing	Type and operating mode of transducer
	Signal Conditioning	Bias regulation, Type and order of filter

A/D converter	Sampling	Sampling frequency
	Quantization	Bit resolution

Serializer/Encoder	Encoder	Data encoding

Which of these knobs is the best to use depends on the target optimization metric or on the specific type of sensed data. Moreover, alterations on a given block also affect downstream components. As a simple example, reducing the A/D bit resolution of an accelerometer from the typical 11- or 12-bit to 8-bit also speeds up and reduces the energy for data transmission [10].

### 2.2 Signal and information content

**Figure 2:**
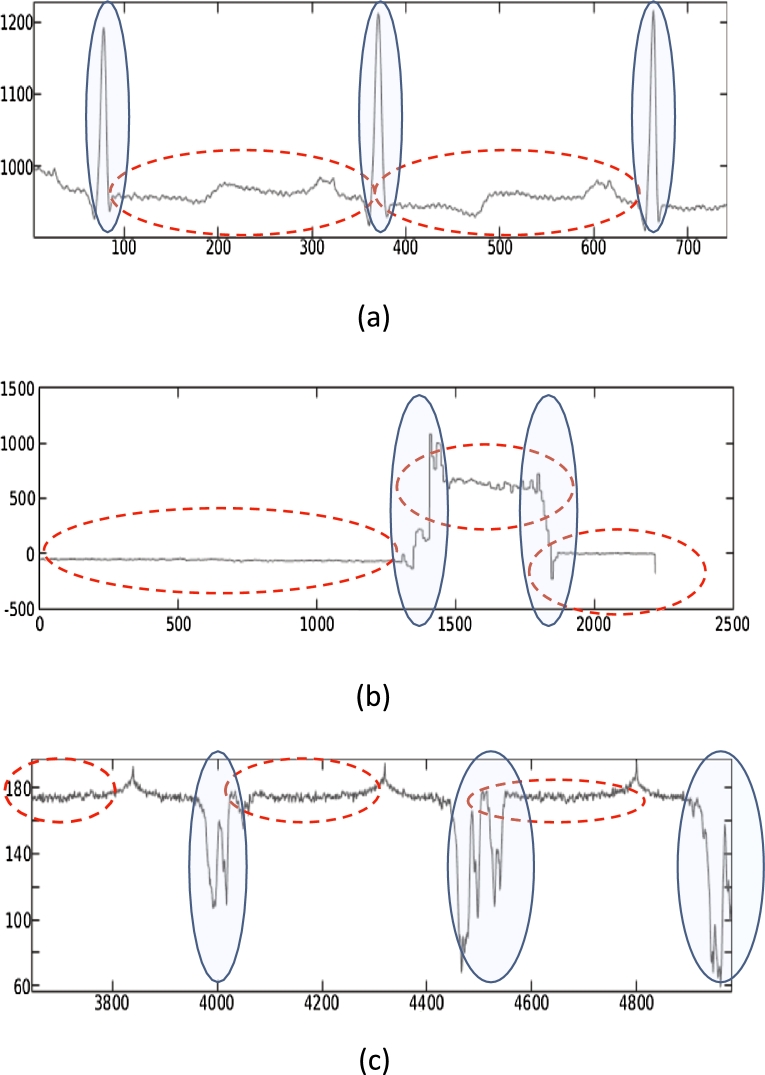
Signal samples from three different sensors: ECG (a), accelerometer (b), and camera (c).

The energy benefits arising from signal approximation during data acquisition (described in Section 2.3) have an impact that depends on the nature of the signals and on the application which uses them. As observed in various previous works [7], [8], [9], [10], [11], a common characteristic of sensor data is the *burstiness* of the signals, i.e. the fact that variations are concentrated in short time windows. This phenomenon is shown in Fig. 2 for three sensed signals: from and ECG sensor (a), from an accelerometer (b), and from an image sensor (c). An observation done in many previous works [8], [10], [11] is that the “relevant” data tend to be localized where variations occur. In the case of the ECG, spikes in the signal correspond to heart pulses, while almost-constant sections correspond to the interval between two heart beats. Similarly, a relatively constant accelerometer signal corresponds to a still device, while sudden value changes correspond to movements. Finally, variations in the grayscale or RGB pixels transmitted by a camera correspond to image features such as edges and lines, whereas constant sections correspond to uniform or slowly-varying colors, which convey less information. This property can be considered as a *variable temporal correlation* of the signals, and is exhibited by the majority of sensors, as detailed in [9].

Intuitively, this characteristic of sensed signals could be leveraged to reduce the impact of approximations resulting from the application of the knobs listed in Table 1. In particular, by approximating more aggressively the regions with high correlation (dotted ovals in figure) and less aggressively (or not at all) the remaining ones, the impact of approximations on downstream tasks (such as a ML classification algorithm) could be reduced, assuming that the *features* extracted and processed by these algorithms are related for instance to image lines, accelerometer movements or ECG pulses. In our experiments, we verify this intuition by comparing two different types of approximation, one focusing only on high-correlation areas of a signal (*smoothing*, see Section 2.3), and the other applying approximations on all samples indistinctly (*rounding*). We show that, contrary to expectations, the former does not yield quantifiably better ML accuracy for a given approximation level.

### 2.3 Smart sensors approximations

As mentioned in Section 2.1, there are multiple points within a data acquisition chain where data approximations can be leveraged in order to achieve energy reductions (see Table 1). Therefore, before focusing on one specific type of approximation used for our practical experiments, we describe the general underlying principles of data acquisition approximations, and how these impact power and energy consumption. In abstract terms, there are two fundamental signal alterations implemented in smart sensors that can impact the power and/or energy, which we call *rounding* and *smoothing*.

The first family of approaches is based on rounding samples according to predefined quantization intervals, i.e. approximating *over values*. For instance, rounding to multiples of 16 means that any value between 8 and 23 will be approximated as 16, whereas values between 24 and 47 will be approximated as 32, and so on.

Rounding can be physically implemented directly when quantizing the analog signal, i.e. by reducing the resolution of the A/D, as shown in [35]. Common A/D types, such as those based on the Successive Approximation Register (SAR) principle, compute each digital output bit sequentially starting from the MSB. For these components, a smaller output resolution results in a faster conversion and in a consequent reduction of the overall energy consumption [9]. Alternatively, rounding can also be implemented during the transmission of digital data, as detailed in Section 3. [7].

With the term *smoothing*, we refer instead to the principle of *eliminating small variations between consecutive values*, i.e. considering similar consecutive values as identical. With respect to rounding, which quantizes *all* values to “bins”, smoothing only joins similar *consecutive samples* (e.g. nearby pixels in case of a camera, or samples for an accelerator). Smoothing reduces energy because it is equivalent to approximating a signal *over time*: if two similar signals are approximated as identical, there is no need to store them, transmit them, or process them twice.

Both rounding and smoothing *distort* the input signal, the former by altering its amplitude non-linearly, and the latter its frequency components. As for rounding, smoothing can be implemented either when sampling the analog signal, by reducing the sampling frequency when data are similar, [6], [30], [31], or when transmitting digital data, e.g. by only sending one datum of each group that is approximated as identical, as detailed in the next section [8], [10].

In the rest of the paper, we concentrate on data transmission approximations, both because of their relevance for energy consumption, and because they allow to easily implement both rounding and smoothing approaches.

## 3 Approximations in sensor data transmission

### 3.1 Energy consumption in serial buses

Serial buses are a de-facto standard for interconnecting off-chip I/O peripherals in embedded digital systems such as most IoT edge devices [34]. Even if consisting of few physical wires, off-chip serial buses can still be significant contributors to the total energy budget of a device. In fact, these buses are typically implemented as PCB traces (e.g., microstrips), whose capacitances are orders of magnitude larger than those of on-chip interconnects. As a consequence, the energy consumption per unit length of a PCB trace is in the order of 1-2 pJ/bit/inch [36], and considering that a PCB trace can span several centimeters, the transmission of a *single bit* can require up to ≈10 pJ. For comparison, a small 32-bit micro-controller (MCU) for sensor-based systems can have active currents in the order of 50-100 μA/MHz, translating to ≈10 pJ/instruction for typical operating frequencies [37]. Therefore, the transmission of each *single bit* on an off-chip serial bus consumes an energy comparable to the execution of one 32-bit instruction on a MCU.

To estimate their energy consumption, off-chip serial connections can be modeled, in first approximation, as purely capacitive channels [36], [38]. Under this model, all power dissipation occurs in correspondence of electrical level changes, i.e., it coincides with the dynamic power, and can be computed as:

(1) $$P_{chan}=P_{dyn}=\alpha C_{tot}V_{swing}^{2}f$$

where $C_{tot}$ is the total load capacitance, including line driver, pin and wire, $V_{swing}$ is the voltage swing between electrical levels, and *f* is the transmission frequency. Finally, α∈[0,1] is the switching probability factor, that accounts for the probability of a level transition in a given clock cycle.

### 3.2 Approximate bus encodings

Bus encodings for smart sensors [7], [8], [9], [10], [11], [36], [38] obtain energy savings by reducing *α* in (1). In other words, they attempt to minimize the *transition count* (TC), i.e. the number of logic-value changes seen on the bus. Transitions are generated by adjacent bits with opposite logic values, either within a word (*intra-word*) or among subsequent words (*inter-word*).

The importance of serial off-chip connections in modern embedded and IoT computing systems has generated quite a vast literature on serial bus encodings. Older solutions are lossless, i.e. they do not exploit data approximations [38]. More recently, approximate serial encodings, which trade-off greater energy (i.e. TC) reductions for small errors in the decoded data, have started being investigated [7], [8], [9], [10], [11], [36]. One of the first efforts in this sense is described in [36], where the authors propose an encoding called Rake, which heuristically inverts the logic value of some bits within a word, in order to generate long sequences of 1s or 0s, and thus reduce the TC. Inversions are performed under a maximum error constraint, in order to balance power saving and data fidelity. More recently, three other encodings called Approximate Differential Encoding (ADE) [9], Serial T0 (ST0) [10] and Axserbus [11] have been proposed, all of which outperform Rake. Interestingly, ADE implements a *rounding* approximation, whereas ST0 and Axserbus perform *smoothing*. Therefore, we decided to focus on these three encodings, as they offer us a simple way to compare the aforementioned generic approximation strategies. Each of them is described in detail in the next subsections.

#### 3.2.1 ADE

ADE, first introduced in [7], is the approximate extension of so-called Differential Encoding (DE). The original DE exploits the *burstiness* of sensor signals by constructing code-words as the bitwise difference (i.e. Hamming distance) between consecutive samples, i.e.:

(2) $$B_{i}[t]=b_{i}[t]⊕b_{i}[t-1],\;\forall i\in [1,n]$$

where ⊕ indicates the binary XOR operator, $b_{i}[t]$ is the i-th bit of the input word at time *t*, and $B_{i}[t]$ is the corresponding bit of the DE codeword [38]. DE decoding is implemented as:

(3) $${\overset{ˆ}{b}}_{i}[t]=B_{i}[t]⊕b_{i}[t-1],\;\forall i\in [1,n]$$

DE yields TC reductions because correlated samples tend to generate Hamming distances with long constant sequences in the Most Significant Bits (MSBs), as explained in [38].

On top of DE, ADE adds a *rounding* approximation, simply obtained by *saturating* some of the Least Significant Bits (LSBs). Calling *n* the bit-width of the input samples and *l* the number of saturated LSBs, ADE encoding works as follows:

(4) $$B_{i}[t]=b_{l+1}[t]⊕b_{l+1}[t-1],\;\forall i\in [1,l]B_{i}[t]=b_{i}[t]⊕b_{i}[t-1],\;\forall i\in [l+1,n]$$

The maximum error introduced by ADE for integer data representations can be computed as $E_{MAX}=2^{l}-1$. ADE decoding is identical to DE. Rounding has the effect of reducing the TC on LSBs, hence further improving the total energy savings compared to standard DE. For a detailed discussion on ADE and its possible variants we refer the reader to [9].

#### 3.2.2 Serial T0

ST0, first introduced in [8], performs approximations only in the high-temporal-correlation regions of a signal, by means of a smoothing approach. The underlying principle of this encoding is that highly-correlated data provide little information, but in most cases compose most of the signal (see Fig. 2). Therefore, the idea is to transmit similar consecutive samples with the minimum possible energy consumption, which corresponds to using a 0-TC pattern, while sending all other words on the bus unaltered. Formally, ST0 builds codewords as follows:

(5) $$B^{\left(t\right)}=\left\{ \begin{array}{cc} \text{0-TC pattern} & \text{if }\|b[t]-b[t^{\prime }]\|\leq T_{h}\\ b[t] & \text{otherwise} \end{array}\right.$$

where $T_{h}$ represents a tunable *maximum error threshold* and $t^{\prime }$ is the index of the last sample that was directly sent on the bus (without approximations). The 0-TC pattern is simply a *n*-bit sequence of logic-1s; the reader is referred to [10] for a detailed analysis of this encoding.

ST0 decoding is implemented as follows:

(6) $$\overset{ˆ}{b}[t]=\left\{ \begin{array}{cc} b[t^{\prime }] & \text{if }B[t]=\text{0-TC pattern}\\ B[t] & \text{otherwise} \end{array}\right.$$

The received codeword is simply copied to the output, except for the 0-TC pattern. In that case, the decoder assumes as output the value of the last valid word (non 0-TC) received. Evidently, ST0 implements smoothing, i.e. repeats the previous sample rather than transmitting a new one, whenever the difference between the two is smaller than $T_{h}$.

#### 3.2.3 Axserbus

Axserbus, introduced in [11] takes on the idea of ST0 and extends it to build a more flexible encoding, supporting a 2-level smoothing. Indeed, this solution uses two smoothing thresholds: $T_{h,0}$ and $T_{h,m}>T_{h,0}$. Furthermore, the encoder also takes into account the residual error from previous transmissions, in order to avoid error accumulation, thus replacing $b[t]-b[t^{\prime }]$ in (5) with $\Delta =(b[t]-b[t^{\prime }])+(b[t]-\overset{ˆ}{b}[t])$, where $\overset{ˆ}{b}[t]$ is the decoded word at step *t*.

When $\Delta <T_{h,0}$, the encoding simply uses the same scheme of (5), i.e. a 0-TC pattern is transmitted, which is interpreted by the decoder as “repeat the previous sample”. In contrast, when $T_{h,0}<\Delta <=T_{h,m}$, a 1-TC pattern is used. Specifically, Δ values between $T_{h,0}$ and $T_{h,m}$ are first approximated in “bins” as:

(7) $$\Delta^{\prime }=2^{\left\lfloor \log_{2}\|\Delta \|\right\rfloor }+2^{\left\lfloor \log_{2}\lfloor \|\Delta /2\|\rfloor \right\rfloor }$$

i.e. each word between $2^{r}$ and $2^{r+1}$ is approximated as the median of the range. Then, $\Delta^{\prime }$ is encoded as a run of l−r 1s followed by *r* 0s, or the opposite if Δ is negative. For example, $\Delta^{\prime }=3$, which corresponds to r=2 is encoded as 11111100 for 8 bit samples. Finally, if $\Delta >T_{h,m}$, the word is transmitted as-is, as long as it naturally generates a TC≥2. Otherwise, one or two LSBs are flipped, in order to avoid that the decoder “confuses” a normal 1-TC word with one of the special patterns described above. Rather than completely eliminating differences or transmitting accurately (as done by ST0), the 2-level scheme of Axserbus allows a more gradual smoothing, in which intermediate differences are still approximated but not eliminated. More details on this solution can be found in [11].

## 4 Impact of approximations on machine learning tasks

The synergy between approximate computing and machine learning is well-studied in the literature, especially for deep neural networks. However, the great majority of papers only study the effects of adding approximations in the *processing* phase of a ML model, i.e. in the computations performed while running a classification [2], [3]. One of the most common approximations of this kind consists of the quantization [20], [21], [22], [23], [24] or binarization [25], [26] of model parameters and intermediate data. The elimination of low-significance connections in deep neural networks (so-called pruning) is another popular form of ML approximation [27], [28], [29].

Approximations of ML model inputs have also been studied, although less extensively [18], [19]. However, the types of alterations considered in those works are very different from those added by actual smart sensors. For instance, the work in [18] tests the impact of four image alterations, i.e. blurring, Gaussian noise addition, contrast reduction and JPEG compression, on the classification accuracy of a Convolutional Neural Network (CNN) for computer vision applications. In [19], the same type of ML model is tested against a variety of image alterations including shot and impulse noise, pixelation, and many others. These works do not relate the transformations tested to a specific approximation performed by a smart sensor, and indeed, this is not always possible. While the effect of *rounding* can be approximated by adding uniform noise on LSBs [8], *smoothing* approximations like those performed by ST0 and Axserbus cannot be easily modeled by a noise distribution, nor by a standard spatial filter. Moreover, modern compression algorithms are also not representative of the approximations introduced during data acquisition. Indeed, the former typically analyze an image globally before “approximating it” in order to reduce storage size. In contrast, alterations added by energy-efficient smart sensors are based on simple *local* algorithms that consider one or a few nearby pixel values in order to keep the energy cost for analysis as low as possible [7], [8], [9], [10], [11]. As anticipated in Section 2.3, these local alterations introduce peculiar types of distortion in the data, which are not easily modeled with standard signal processing techniques.

The recent work of [35] considers, among other types of noise and failure-induced errors, the impact of rounding approximations introduced by reducing the A/D conversion precision on the accuracy of several machine learning models. However, the authors do not compare the latter with smoothing approximations, and focus on a single activity recognition use case.

Other works have studied the effect of *label noise*, i.e. incorrect labeling of training data, on the accuracy of deep learning models [39]. Again, this is a very different target from ours, since label noise affects training (while we focus on the inference phase) and is produced by human errors, not by a data acquisition approximation.

Finally, a vast literature has studied *adversarial* alterations of the input of a ML model, in order to force a misclassification [40], [41], [42]. While these types of alterations are very important for ML security and intellectual property protection, being specifically tailored to induce errors in the model, they are clearly not representatives of the approximations introduced by smart sensors.

In summary, despite the large number of previous works that have studied the relation between approximation and ML accuracy, to the best of our knowledge, ours is the first to explicitly compare the effect of simple rounding and smoothing approximations introduced by energy-efficient smart sensors.

## 5 Experimental results

### 5.1 Setup

We tested the impact of ADE, ST0 and Axserbus on two different ML tasks: image classification and activity recognition based on inertial sensors. Notice that, although ADE, ST0 and Axserbus have already been compared in [11], those experiments were based on generic image quality metrics (e.g. the Peak Signal-to-Noise-Ratio - PSNR). The effect of these three encodings on ML classification accuracy has never been assessed.

The two target tasks have been selected due to their relevance in several edge ML applications [2], [3] and to their different characteristics. Indeed, image classification is a high-data-rate application, in which a smart sensor (i.e. camera) has to sample and transmit large amounts of data (in the order of kilobytes or megabytes per image) at high-frequency, hence incurring a large power consumption in off-chip buses (see Equation (1)) [10]. At the same time, this type of task typically requires a complex deep learning model to achieve high accuracy, with a consequent high consumption in the processing part of the system. In contrast, inertial sensors data are smaller in size, but can be reliably classified using much simpler classic ML models such as k-Nearest Neighbors (k-NN) and Support Vector Machines (SVM), with a much lower computational burden. In both cases, off-chip buses can be relevant contributors to the total energy of the system.

For image classification, we tested the three approximate encodings against two state-of-the-art Convolutional Neural Networks (CNNs), i.e. MobileNetV2 [43] and InceptionV3 [44]. MobilenetV2 is specifically tailored at executing inference on mobile and embedded devices, thanks to the use of depthwise separable convolutions and other model optimizations to reduce complexity, whereas InceptionV3 is significantly more complex (≈ 6x larger weights size) but also significantly more accurate. These two CNNs allow us to assess whether a larger model is indeed more resilient to input noise. For both networks, we used the implementations made available by the Keras deep learning framework [45], which are provided pre-trained on the ImageNet dataset [46]. All tests have been performed on the original ImageNet validation set, which contains 50000 images, assuming a 24-bit RGB representation for image pixels.

For activity recognition, we used the UniMiB SHAR dataset [47], which contains around 11000 3-axis accelerometer patterns grouped in 17 classes of activity. We have split the dataset randomly using 70% of patterns for model training and the remaining 30% for validation. Data are provided as floats, which we have converted to 16-bit fixed-point, to simulate the typical data format of a commercial accelerometer [48]. To classify these data, we built 3 simple classifiers similar to those used in [47]. Specifically, we used a k-NN with k=1, a SVM with Radial Basis Function (RBF) kernel, and a simple 1-dimensional CNN with two convolutional layers (3x3 kernels, 64 channels, Rectified Linear Unit - ReLU activation) followed by a max pooling layer with pool size =2 and by two dense layers with an hidden size of 100. With this second set of experiments, we can therefore assess weather a deep learning model (the 1-D CNN) is more resilient to smart sensor approximations compared to a much less computationally intensive k-NN or SVM. A summary of our experiments is shown in Table 2.

**Table 2:** Experiments summary.

Dataset	Classifier
ImageNet [46]	MobileNetV2
	InceptionV3

UniMiB SHAR [47]	k-NN with *k* = 1
	SVM with RBF
	1D-CNN

We used a Python library to simulate the encoding of all data with the three bus encodings. We repeated the simulated transmission multiple times, varying the parameters of each encoding that control the amount of approximation, i.e. *l* for ADE, $T_{h}$ for ST0 and $T_{h,0}$ and $T_{h,m}$ for Axserbus. We then ran the ML classification on the decoded data and compared the results with those obtained on not approximated data. Since Axserbus has two free parameters, we performed a grid-search on both and then extracted the Pareto-optimal combinations.

### 5.2 Image classification results

**Figure 3:**
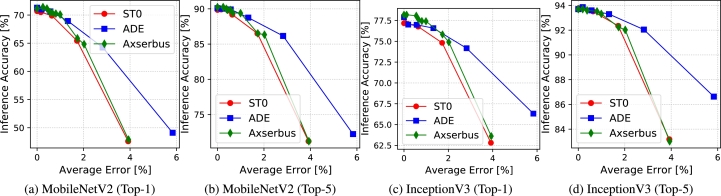
Accuracy versus average bus encoding error for image classification.

**Figure 4:**
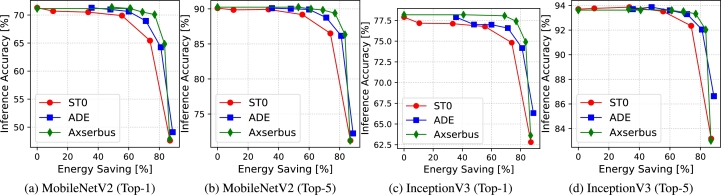
Accuracy versus bus energy saving for image classification.

Results for the image classification task are reported in Figure 3, Figure 4. Both sets of plots report the accuracy of ML classification on the y axis. Both Top-1 and Top-5 accuracy are reported, since both metrics are commonly used in this domain.^1^ On the x axis, Fig. 3 shows the average error magnitude on each decoded word (i.e. pixel) whereas Fig. 4 shows the actual energy saving on the serial bus, based on the model of (1). Both measures are reported in percentage. The leftmost points of all curves correspond to no approximation, so the corresponding accuracy is the nominal one obtained on the unaltered ImageNet validation set.

The two sets of plots show two different aspects of the comparison. Fig. 3 shows the impact of a generic average *rounding* or *smoothing* error on the accuracy of the two CNNs. This dependency is roughly independent of where the approximation is performed and therefore helps in comparing the two generic approximation strategies. In contrast, the energy results in Fig. 4 are specific to serial bus approximations, and strongly depend on the implementation details of ADE, ST0 and Axserbus.

These results offer very interesting insights on the two types of approximations. In fact, Fig. 3 shows that rounding is almost always superior to smoothing in the accuracy versus decoding error space, especially for aggressive approximations, i.e. a superior ML accuracy is obtained for a given average error. This is a surprising result, which goes against the intuition of previous work, described in Section 2, that approximating only high-temporal-correlation regions would affect less the features extracted by ML classifiers. In contrast, it appears that rounding, which acts like a uniform noise applied to all input pixels, and is roughly equivalent to input quantization, is better tolerated by the two CNNs compared to the data-dependent approximations introduced by smoothing methods. This can be motivated by the well-known high resilience of CNNs to quantization.

On the accuracy versus energy plane of Fig. 4, results are different. ADE still outperforms ST0 but is almost invariably worse than Axserbus. This difference, however, boils down to the clever implementation details of Axserbus [11] and not to the underlying nature of the performed approximations. Moreover, the quantitative difference between the two is in most cases quite small (e.g. Fig. 4d), which makes ADE still an interesting alternative for approximate serial transmission from a smart camera sensor, especially considering that the silicon area and power consumption of the encoding and decoding hardware in Axserbus is almost 10x larger than that of ADE for the same technology node (see the results in [11] and [9] for a comparison).

Looking at the two figures together also stimulates interesting observations. In fact, ST0 and Axserbus, both based on smoothing, are almost identical in terms of accuracy versus average error, but the latter is definitely superior when considering actual bus energy. Combining this with the superior results of ADE for the same average error, shows that there is probably still a lot of space to design clever serial bus encodings based on rounding, which could even outperform Axserbus in terms of accuracy versus energy. We believe that this is one of the most important conclusions of our study.

Interestingly, Fig. 3 also shows that larger models are indeed more resilient to approximations. For example, looking at the Axserbus curve, a 2% average error on transmitted pixels causes a >5% drop in Top-1 accuracy for MobileNet, whereas the same pixel-level error on Inception only causes a ≈2.5% drop. However, these results should be weighted considering that Inception requires a much larger processing energy to perform a classification. Therefore, the additional resilience to errors in smart sensors is “paid” on the processing side.

Analyzing the results from the point of view of serial transmission energy, we can see that regardless of the metric and CNN model considered, very large savings are possible with a negligible impact on accuracy. With Axserbus, for instance, 68%, 83%, 76% and 77% energy reductions are achieved with less than 1% accuracy drop on MobileNet Top-1 and Top-5, and Inception Top-1 and Top-5 respectively.

### 5.3 Activity recognition results

**Figure 5:**
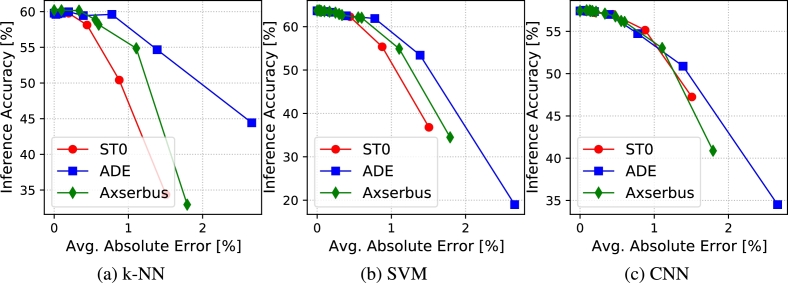
Accuracy versus bus encoding error for activity recognition.

**Figure 6:**
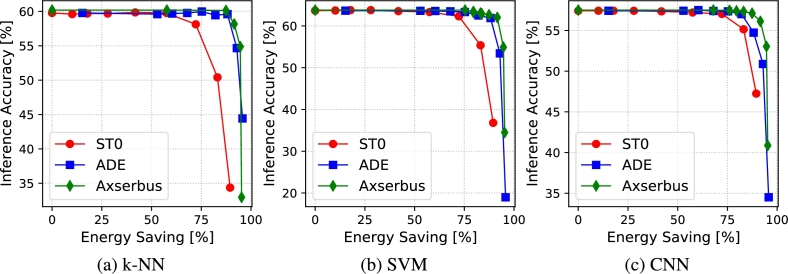
Accuracy versus bus energy saving for activity recognition.

Figure 5, Figure 6 show analogous results of Figure 3, Figure 4 but for the activity recognition application. In this case, we only report results relative to the Top-1 accuracy, since the dataset only includes 17 classes. For such a small number of classes, the Top-5 accuracy, although valid in principle, is not a very informative metric, as even a random classifier put the correct class in the top-5 list ≈30% of the times.

All observations done in Section 5.2 remain valid also for this second application, despite the different nature of the serially transmitted data and of the ML classifiers used. This confirms that those observations are not linked to the specific use case of image classification, but remain valid for different categories of sensor data.

The savings achieved for a given accuracy drop are even higher in this case, due to the simpler classification task. With Axserbus, bus energy is reduced by 87%, 87% and 84% with a <1% accuracy drop on the CNN, k-NN and SVM respectively. Also, the difference between Axserbus and ADE is smaller on this stask, with the latter achieving 82%, 88% and 75% in the same conditions.

Finally, a last important observation is that classic ML classifiers (k-NN and SVM) show both a similar accuracy in absence of data alterations and a similar resilience to approximations compared to the CNN. This shows that, at least for a simple task like human activity recognition based on accelerometer data, even a simple classifier like a SVM, which requires significantly less processing to perform a classification, can still be used in conjunction with aggressive data acquisition approximations. This result is apparently in contrast with the one obtained in [35], where the authors found that deep-learning models significantly outperformed classic ML approaches in presence of A/D quantization. However, this is probably due to the different model size and dataset considered. In fact, the authors of [35] used a 5-layer dense neural network with a total of approximately 3M parameters, whereas our smaller CNN only includes ≈0.5 M weights. As detailed in the image classification section, a larger model size is likely to yield a higher error resilience, but may result in a too high computational complexity for a simple activity recognition task. Similarly, the dataset used in our experiments includes a smaller number of features (i.e. only the 3 accelerometer axes readings versus a total of 45 readings in [35]). The higher amount of redundancy in the input data is likely to give the deep learning model of [35] more opportunities to cope with the reduced precision, following a well known trade-off of Approximate Computing [16]. Overall, we argue that our results analyze the error resilience of human activity recognition based on inertial data and ML models from a different perspective compared to [35]. In particular, we focus specifically on a use case that could be implemented in a low-power edge device (e.g. a wearable), which can only collect a limited quantity of data and must use simple ML models for classification.

## 6 Conclusions

We have presented an analysis of the impact of the two most common types of smart sensor approximations on the accuracy of two different ML tasks. Our experiments have shown that, contrarily to intuition, the category which we denoted as *rounding* affects less the performance of ML classifiers compared to *smoothing*. In the specific case of energy-efficient approximate serial data transmission, however, the availability of advanced encoding techniques based on smoothing makes the two categories comparable, with Axserbus [11] achieving the best results. Nonetheless, the rounding-based ADE [9] still achieves comparable energy savings for a given accuracy level on most experiments, which make it an interesting alternative to consider by system designers, especially given that it permits a smaller and more efficient hardware implementation of encoding and decoding.

Finally, we have also shown that, for simple tasks like activity recognition, classic solutions like SVM and k-NN offer similar resilience to data acquisition approximations compared to a CNN. In contrast, for complex image classification tasks, very large deep learning models like Inception are indeed more resilient to data alterations, although this advantage comes at the cost of an increased processing energy.

Motivated by these findings, our future work will focus on the development of more advanced rounding-based approximations, not limited only to the serial transmission of data, but extended to the entire sensing chain, in order to fully exploit the resilience of ML models to this type of approximation. Moreover, we also plan on extending our experiments to other use cases, such as bio-signals processing.

## CRediT authorship contribution statement

**Daniele Jahier Pagliari:** Methodology, Data curation, Software, Visualization, Validation, Writing – Original draft preparation. **Massimo Poncino:** Conceptualization, Methodology, Writing – Reviewing and Editing, Supervision.

## Declarations

### Author contribution statement

D.J. Pagliari: Conceived and designed the experiments; Performed the experiments; Analyzed and interpreted the data; Wrote the paper.

M. Poncino: Conceived and designed the experiments; Analyzed and interpreted the data; Wrote the paper.

### Funding statement

This research did not receive any specific grant from funding agencies in the public, commercial, or not-for-profit sectors.

### Data availability statement

Data included in article/supplementary material/referenced in article.

### Declaration of interests statement

The authors declare no conflict of interest.

### Additional information

No additional information is available for this paper.

## Footnotes

1 When computing the Top-1 accuracy, a classification is considered correct only if the class label deemed as most probable by the CNN corresponds to the ground truth. In contrast, for the Top-5 accuracy, the correct label should be among the 5 most probable ones according to the network.
